# ATG9A-dependent, LC3-independent autophagy curbs the immune system to protect against disease

**DOI:** 10.1080/27694127.2026.2614147

**Published:** 2026-01-12

**Authors:** Dario Priem, Mathieu JM Bertrand

**Affiliations:** a Center for Inflammation Research, VIB, Ghent, Belgium; b Department of Biomedical Molecular Biology, Ghent University, Ghent, Belgium

**Keywords:** ATG9A, cell death, cGAS, inflammation, inflammatory skin disease, LC3-independent autophagy, nucleic acid immunity, STING, TNF, ZBP1

## Abstract

Selective autophagy is generally believed to require the conjugation of microtubule associated protein 1 light chain 3 (LC3) proteins (or other autophagy-related 8 [ATG8] family members) on the inner phagophore leaflet to enable the recruitment of cargo-bound selective autophagy receptors. However, this paradigm is challenged by the discovery that cytosolic cargoes can still be selectively targeted by phagophores even in the absence of LC3 proteins. In a recent study published in *Immunity*, we discovered that ATG9A-dependent, LC3-independent autophagy facilitates the degradation of multiple inflammatory signaling complexes to prevent an inflammatory skin disease.

Inflammation is the first response of the immune system to invading microbial pathogens or tissue injury. Specialized innate immune receptors react to these insults by inducing the expression of various pro-inflammatory mediators, which collectively attract specialized immune cells to the site of infection or injury, promote the elimination of infected/damaged cells, and finally initiate the healing process. Besides activating gene expression, several innate immune receptors also developed the capacity to induce programmed cell death, either apoptosis, necroptosis or pyroptosis. Cell demise not only helps clearing damaged or infected cells but also indirectly promotes inflammation by releasing intracellular danger molecules into the environment, which activate immune receptors on neighboring cells. Although (cell death-driven) inflammation is essential to protect the body from insults, dysregulation of these pathways can turn inflammation into a maladaptive response and triggers the development of numerous acute and chronic inflammatory diseases. To prevent this detrimental shift, cells employ various regulatory mechanisms that restrain excessive inflammation and cell death, thereby safeguarding tissue integrity. One of those mechanisms is autophagy, which plays a major role in terminating immune responses by promoting the selective degradation of immune signaling complexes.

A recent view is that selective macro-autophagy is mediated by selective autophagy receptors that recognize autophagic cargoes and recruit the autophagy-related machinery to induce the local formation of a phagophore. The microtubule associated protein 1 light chain 3 (LC3) molecules (or other autophagy-related 8 [ATG8] family members) anchored to the inner phagophore leaflet serve as docking sites for those selective autophagy receptors, thereby facilitating the subsequent encapsulation of the receptor-bound cargoes into autophagosomes. Due to the pivotal role of LC3 proteins in this process, genetic targeting of components of the LC3-conjugation systems such as ATG16L1, ATG5 and ATG7, are often used to evaluate the contribution of autophagy to any biological response. Several recent *in vitro* studies have however revealed the existence of additional forms of selective autophagy that can bypass the need for LC3 proteins, and whose physiological importance has consequently been mostly overlooked. During this LC3-independent process, the selective autophagy receptors directly engage the autophagy-initiation machinery (e.g. through RB1 inducible coiled-coil 1 [RB1CC1]/FAK family kinase-interacting protein of 200 kDa [FIP200] binding) to locally generate an autophagosome around the cargo. Mice constitutively lacking autophagy initiation proteins such as ATG9A or RB1CC1 die *in utero*, whereas mice deficient for any component of the LC3-conjugation systems survive until birth. These findings reveal that LC3 is not absolutely required for macro-autophagy and highlight the vital contribution of LC3-independent macro-autophagy to mammalian physiology.

In our recent work published in *Immunity* ^[1]^, we aimed to study the potential anti-inflammatory properties of LC3-independent autophagy in adult mice. Due to the embryonic lethality of constitutive *atg* knockout mice, we instead generated mice harboring a keratinocyte-specific deletion of *Atg9a* (*Atg9a*^*ΔKer*^ mice) or *Atg16l1* (*Atg16l1*^*ΔKer*^ mice). Mice lacking ATG9A in the skin spontaneously developed a severe inflammatory skin disorder, characterized by back skin lesions, epidermal hyperplasia, compromised skin barrier integrity and systemic inflammation. These mice also displayed a pronounced type I interferon (IFN) response and showed increased levels of keratinocyte cell death. In contrast, *Atg16l1*^*ΔKer*^ mice did not develop any overt inflammatory skin phenotype (neither did *Atg5*^*ΔKer*^ or *Atg7*^*ΔKer*^ mice), pointing toward a prominent role of LC3-independent macro-autophagy in maintaining skin homeostasis.

The further and systematic analysis of the *Atg9a*^*ΔKer*^ mice then allowed us to precisely define the pathogenesis of the disease. We found that ATG9A inhibits three key steps in a self-amplifying inflammatory cascade that prevents pathological skin inflammation. At first, ATG9A suppresses the initiation of the disease by preventing tumor necrosis factor (TNF)-dependent keratinocyte apoptosis. TNF is a master pro-inflammatory cytokine that triggers inflammation either directly by promoting inflammatory gene expression or indirectly by inducing cell death. TNF receptor 1 (TNFR1) activation by TNF leads to the formation of a cytotoxic protein complex called TNFR1 complex II. We found that LC3-independent autophagy prevents TNF-mediated apoptosis by targeting TNFR1 complex II for lysosomal degradation. Cells lacking ATG9A or any other upstream ATG protein, such as RB1CC1, ATG13 and vacuolar sorting protein 34 (VPS34), die in response to TNF, while cells deficient in components of the LC3-conjugation machinery do not. We found that the increased levels of TNF-mediated keratinocyte apoptosis in the *Atg9a*^*ΔKer*^ mice causes local skin inflammation (Figure 1).

**Figure 1.**
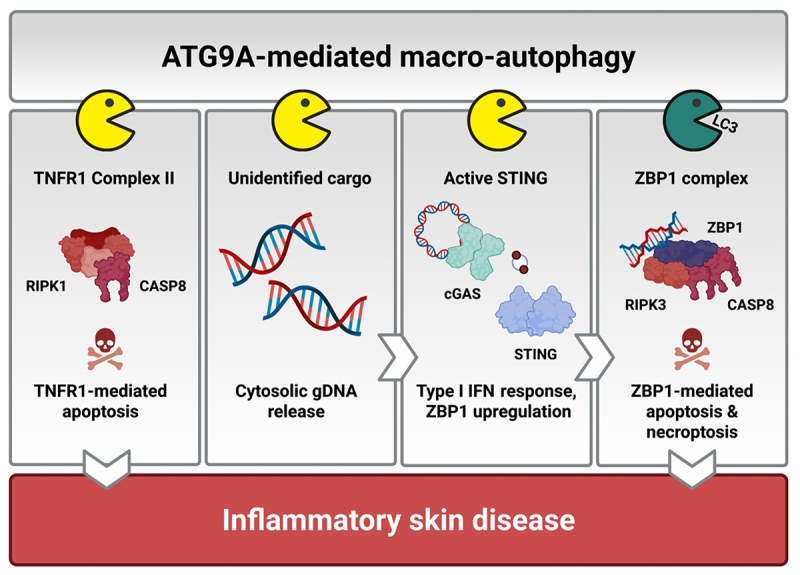
ATG9A-dependent macro-autophagy promotes the degradation of different inflammatory cargoes to prevent pathological skin inflammation. ATG9A-dependent LC3-independent macro-autophagy prevents TNF-mediated keratinocyte apoptosis by promoting the degradation of TNFR1 complex II. Excessive TNF-mediated apoptosis causes local skin inflammation in the *Atg9a*^*ΔKer*^ mice. ATG9A also restricts the TNFR1-mediated cytosolic release of genomic DNA (gDNA), which triggers cGAS activation. In addition, ATG9A inhibits cGAS/STING-dependent type I IFN production by promoting the LC3-independent lysosomal degradation of activated STING. Finally, ATG9A represses ZBP1-mediated apoptosis and necroptosis by detoxifying the ZBP1 complex through canonical LC3-dependent macro-autophagy. Aberrant ZBP1-mediated keratinocyte death induces skin lesion formation and systemic inflammation in the *Atg9a*^*ΔKer*^ mice.

Secondly, we showed that LC3-independent autophagy represses the ability of TNF to trigger cyclic GMP-AMP synthase (cGAS)/stimulator of interferon genes (STING)-mediated type I IFN production. cGAS is a cytosolic innate immune receptor that recognizes double-stranded DNA. Once activated, cGAS engages the adaptor protein STING to promote type I IFN production. We found that ATG9A limits the TNF-dependent cytosolic release of genomic DNA, which triggers cGAS activation (Figure 1). Moreover, we demonstrated that ATG9A additionally represses cGAS/STING signaling by promoting the lysosomal degradation of active STING (Figure 1). Interestingly, ATG16L1 deficiency did not increase the abundance of cGAS ligands, nor did it impact cGAS/STING signaling, indicating that these pathways are specifically repressed by LC3-independent autophagy. Preventing TNF-mediated cGAS/STING activation in *Atg9a*^*ΔKer*^ mice not only resolved the excessive type I IFN production, but also completely prevented the development of skin lesions and rescued the systemic inflammation.

Finally, we demonstrated that ATG9A directly prevents skin lesion formation by suppressing Z-DNA-binding protein 1 (ZBP1)-mediated keratinocyte apoptosis and necroptosis. ZBP1 is an IFN-inducible Z-nucleic acid sensor originally reported to mediate antiviral immunity by promoting necroptosis, a highly inflammatory lytic form of cell death. Besides its role during infection, ZBP1 can also induce sterile inflammation by acting on endogenous Z-nucleic acids produced by the host cells, for instance keratinocytes. Given the pronounced type I IFN signature in the *Atg9a*^*ΔKer*^ mice, keratinocytes from these animals exhibited markedly elevated ZBP1 expression. We found that ATG9A represses ZBP1 cytotoxicity by promoting its lysosomal degradation (Figure 1). The detoxification of ZBP1 proceeds through conventional autophagy, since ATG16L1 deficiency equally sensitized cells to ZBP1-mediated killing. We found that the combination of excessive ZBP1-mediated apoptosis and necroptosis in *Atg9a*^*ΔKer*^ mice drives skin lesion formation and underlies the systemic pathology.

To summarize, our findings show that ATG9A-dependent autophagy acts as a master regulator of skin homeostasis by suppressing three interconnected inflammatory pathways: (I) it prevents TNFR1 complex II-mediated keratinocyte apoptosis, (II) it restricts TNFR1-driven cGAS/STING-dependent type I IFN production, and (III) it subsequently represses ZBP1-mediated keratinocyte death. Our data indicates that part of ATG9A’s immunosuppressive functions are mediated by LC3-independent autophagy, underscoring the previously underappreciated physiological importance of this newly discovered macro-autophagy pathway. Indeed, our data exemplifies that we should be cautious when addressing the biological requirement of autophagy by only genetically targeting components of the LC3 conjugation machinery. Given the central importance of protein degradation in preventing excessive inflammatory signaling, it will be important to identify which other inflammatory pathways are regulated by LC3-independent macro-autophagy (or other alternative forms of autophagy) and to determine how defects in these processes contribute to disease.

## Abbreviations

ATG: autophagy-related;
cGAS: cyclic GMP-AMP synthase
DNA: deoxyribonucleic acid
FIP200: FAK family kinase-interacting protein of 200 kDa
IFN: interferon
LC3: microtubule-associated protein 1 light chain 3
RB1CC1: RB1 inducible coiled-coil 1
STING: stimulator of interferon genes
TNF: tumor necrosis factor
TNFR1: TNF receptor 1
VPS34: vacuolar sorting protein 34
ZBP1: Z-DNA-binding protein 1

## Data Availability

Data sharing is not applicable to this article as no data were created or analyzed in this study.

## References

[1] Priem D, Huyghe J, Gilbert B, et al. ATG9A-mediated autophagy prevents inflammatory skin disease by limiting TNFR1-driven STING activation and ZBP1-dependent cell death. Immunity. 2025;58(12):2972–2988.e6. doi: 10.1016/j.immuni.2025.09.019 41118755

